# Clinical importance of the antibiotic regimen in transrectal ultrasound-guided biopsy: quinolone versus cephalosporin

**DOI:** 10.1186/s12894-016-0169-z

**Published:** 2016-08-24

**Authors:** Jung Keun Lee, Sangchul Lee, Sung Kyu Hong, Seok-Soo Byun, Sang Eun Lee

**Affiliations:** 1 Department of Urology, Seoul National University Bundang Hospital, 166, Gumi-ro, Bundang-gu, Seongnam, Gyunggi-do 463-707 South Korea; 2 Department of Urology, College of Medicine, Seoul National University, 103, Daehak-ro, Jongno-gu, Seoul, 110-799 South Korea

**Keywords:** Antibiotics, Biopsy, Infection, Prostate, Transrectal ultrasound

## Abstract

**Background:**

Quinolone is recommended as an antimicrobial prophylaxis to prevent infectious complication after transrectal ultrasound-guided biopsy, but the increased appearance of quinolone-resistant organism has raised concerns about the efficacy of quinolone. The current study was performed to evaluate various clinical factors including antimicrobial regimens associated with infectious complication after transrectal ultrasound-guided prostate biopsy.

**Methods:**

The medical records of 5215 patients who underwent a multicore transrectal ultrasound-guided prostate biopsy between May 2003 and January 2013 at our institution were reviewed. We analyzed clinical variables including prostate-specific antigen, International Prostate Symptom Score, antimicrobial regimen, prostate size, and number of biopsy cores. Univariate and multivariate logistic regression analyses of infection-related hospitalization after prostate biopsy were performed.

**Results:**

The mean age and median prostate-specific antigen of the entire cohort were 66 years and 6.4 ng/ml, respectively. Twenty-eight (0.54 %) patients developed an infectious complication after prostate biopsy that required hospitalization. Patients who received prophylactic quinolone showed a higher infectious hospitalization rate than patients who received prophylactic third-generation cephalosporin (1.5 vs. 0.3 %; *p* < 0.001). Multivariate logistic regression analysis demonstrated that the International Prostate Symptom Score (odds ratio = 3.18, 95 % confidence interval 1.24–8.13, *p* = 0.016) and the use of third-generation cephalosporin (odds ratio = 0.21, 95 % confidence interval 0.10–0.44, *p* < 0.001) were independent predictors of infection-related hospitalization after prostate biopsy.

**Conclusion:**

With the emergence of quinolone-resistant microorganisms, third-generation cephalosporin may effectively reduce the risk of infectious complications after transrectal ultrasound-guided prostate biopsy. Severe lower urinary tract symptoms may also be an independent risk factor for infection-related hospitalization after transrectal ultrasound-guided prostate biopsy.

## Background

Transrectal ultrasound-guided prostate biopsy (TRUS-PBx) is the standard procedure for diagnosing prostate cancer, and several million TRUS-PBx procedures are performed annually worldwide [1]. However, one of the most serious complications associated with TRUS-PBx is infection such as urosepsis which can be potentially fatal. The hospital admission rate due to infectious complications after TRUS-PBx has been reported as 0.8–3.6 % of patients [2–4]. Although appropriate antimicrobial prophylaxis is generally administered, an increased trend in infection-related hospitalization after TRUS-PBx has recently been reported [5–7]. Potential risk factors for infectious complications after TRUS-PBx included a high comorbidity index, uncontrolled diabetes mellitus, large prostate, untreated bacteriuria, an indwelling urinary catheter, bladder stones, and a recent history of urinary infection such as bacterial prostatitis [8, 9]. The most important clinical risk factor among many is the presence of antibiotic-resistant pathogens in the rectal flora [6, 8, 9].

Antibiotic regimens play a key role in the management of TRUS-PBx and oral fluoroquinolones are used extensively for standard antibiotic prophylaxis [10, 11]. However, fluoroquinolone-resistant species are increasing, so the frequency of infectious complications with these antibiotic-resistant organisms is likely to increase [12–14]. Various antimicrobial prophylactic regimens have been proposed previously to decrease the infectious complication rate after TRUS-PBx [4, 15].

In the present study, we investigated the various clinicopathological factors associated with infectious complications after TRUS-PBx. Our country has an extended national health insurance policy that covers the expenses of an inpatient stay for the entire population [16], so most patients in our facility underwent TRUS-PBx as inpatients. Consequently, we had a unique opportunity to analyze the incidence of infectious complications in patients who were managed by physicians following TRUS-PBx.

## Methods

We retrospectively analyzed the electronic medical records of 5376 patients who underwent a multicore TRUS-PBx after admission to our institution between May 2003 and January 2013. Clinical characteristics including age, body mass index, diabetes mellitus, prostate-specific antigen (PSA), International Prostate Symptom Score (IPSS), the antimicrobial regimen, prostate size, number of biopsies, and TRUS-PBx complications were evaluated. We excluded the patients who were administered other prophylactics (*n* = 77), and patients with insufficient medical data (*n* = 84). Finally, clinicopathological data for 5215 patients were analyzed.

Most TRUS-PBx within our institute were performed in an inpatient setting. The decision to admit a patient was made on the basis of concern of patient or the preference of the physician. Patients provided written informed consent, and underwent a single glycerin enema on the day of the biopsy. Most urine cultures were performed for 1–3 weeks before TRUS-PBx. If the urine culture showed clinically significant growth of bacteria, antibiotics based on the urine culture were prescribed and TRUS-PBx was delayed until the patients had recovered from infection. If no significant growth of bacteria was seen in the urine culture, physician-preferred prophylactic antibiotics were administered intravenously once before biopsy. Third generation cephalosporin was generally the preferred antibiotic in recent years. Between 2008 and 2013, 168 (6 %) patients were given quinolone before biopsy, and 2699 patients (94 %) were given third-generation cephalosporin as prophylactic antibiotics. The quinolone group received a single dose of 400 mg ciprofloxacin or 250 mg leveofloxacin. Third-generation cephalosporin groups received a single dose of 1 g flomoxef, 1 g ceftriaxone, or 1 g ceftizoxime. All patients underwent prophylactic antibiotics with the dose adjusted to renal and hepatic function. Thereafter, all patients received oral antibiotics for 2 days after TRUS-PBx. Two experienced radiologists performed TRUS-PBx, which is a routine, standard multicore biopsy that is performed using local anesthesia. In the inpatient setting, patients stayed in the hospital for 1 day or until the absence of complications such as rectal bleeding or gross hematuria was confirmed. Patients who underwent TRUS-PBx were instructed to go to an emergency room or urologic clinic if they developed a high fever, severe bleeding, or severe lower urinary tract symptoms including urinary retention. All patients were followed up as at outpatients within 2 weeks. Physicians recorded the results of TRUS-PBx and evaluated the complications at the follow-up.

Infection-related hospitalization after TRUS-PBx was defined as a new admission to the hospital or a delaying discharge due to a systemic inflammatory syndrome such as a fever of 37.8 °C (100 °F) or higher within 2 weeks of TRUS-PBx regardless of significant growth of bacteria in a blood culture. Initiating antibiotic therapy was deferred until blood cultures were obtained in the case of infection-related hospitalization after TRUS-PBx. Blood cultures were available in all patients with infectious complications. Although no standard guidelines exist for management of infectious complications after TRUS-PBx, empirical patient-specific antimicrobial therapy was administered, followed by culture-driven antimicrobial therapy if TRUS-PBx-related sepsis occurred.

The primary clinical data were treated as categorical or continuous variables and screened for the entire group of patients. Univariate and multivariate logistic regression analyses of infection-related hospitalization after TRUS-PBx were performed. All *p* values were two-sided, and values <0.05 were considered to be statistically significant. Adjusted odds ratios (OR) and their 95 % confidence intervals (CI) were calculated using multiple logistic regression analyses. All data analyses were performed using the Statistical Package for the Social Sciences® 18.0 software (SPSS Inc., Chicago, IL, USA).

This study was conducted in compliance with the Helsinki Declaration and approved by our local ethics committee. The reference number of Seoul National University Bundang Hospital Institutional Review Board is B-1407/260-111. The ethics committee did not require informed patient consent because the study was retrospective. Data from medical records were analyzed after anonymization.

## Results

Clinicopathological characteristics of the 5215 TRUS-PBx patients included in the final analysis are shown Table 1. The mean age and median PSA of the entire cohort were 64.7 ± 9.3 years and 6.5 ng/ml (interquartile range, 4.3–10.6 ng/ml), respectively. Of these patients, 19 % were included in the quinolone group and 81 % in the third-generation cephalosporin group. Twenty-eight (0.54 %) patients developed an infectious complication that required hospitalization after TRUS-PBx (Table 1).

**Table 1:** Comparison of clinicopathological features among men who underwent a contemporary multicore prostate biopsy

Variable	Entire cohort
Number of patients	5215
Median age (years)(IQR)	66 (60–71)
Mean BMI (kg/m^2^)	24.3 ± 2.7
Diabetes mellitus (%)	742 (14.2)
Median PSA (ng/mL)(IQR)	6.4 (4.3–10.5)
Mean prostate volume (mL)	45.6 ± 22.5
IPSS (%)	
Mild	1835 (35.2)
Moderate	2439 (46.8)
Severe	941 (18.0)
Year of prostate biopsy (%)	
2003–2007	2348 (45.0)
2008–2013	2867 (55.0)
Antibiotic prophylaxis (%)	
Quinolone	990 (19.0)
Third-generation cephalosporin	4225 (81.0)
Number of biopsy cores (%)	
≤ 12	3470 (66.5)
≥ 13	1745 (33.5)
Pathologic diagnosis after biopsy (%)	
Carcinoma	1780 (31.4)
Prostatitis	438 (8.4)
Others	2.997 (57.5)
Admission for infectious complications (%)	28 (0.54)

Univariate and multivariate logistic regression analyses of infection-related hospitalization after TRUS-PBx are shown in Table 2. Univariate analyses indicated that a prophylactic antibiotics regimen based on third-generation cephalosporin (OR = 0.20, 95 % CI 0.10–0.42, *p* < 0.001) and severe IPSS (OR = 3.37, 95 % CI 1.32–8.60, *p* = 0.011) were independent predictors of infection-related hospitalization after TRUS-PBx (Table 2). Multivariate analysis of 4932 patients indicated that third-generation cephalosporin (OR = 0.21, 95 % CI 0.10–0.44, *p* < 0.001) and severe IPSS (OR = 3.18, 95 % CI 1.24–8.13, *p* = 0.016) were also independent predictors of infection-related hospitalization after TRUS-PBx (Table 2).

**Table 2:** Univariate and multivariate analyses of infection-related hospitalization after prostate biopsy

Variable	Univariate		Multivariate^a^	
	OR (95 % CI)	*p* value	OR (95 % CI)	*p* value
Age				
< 70	1.0			
≥ 70	0.83 (0.38–1.89)	0.655		
BMI	0.99 (0.86–1.13)	0.825		
Diabetes mellitus	0.72 (0.22–2.40)	0.595		
Prostate volume	1.00 (0.98–1.02)	0.941		
IPSS				
Mild	1.0		1.0	
Moderate	0.36 (0.36–2.60)	0.947	0.94 (0.35–2.52)	0.936
Severe	3.37 (1.32–8.60)	0.011	3.18 (1.24–8.13)	0.016
Year of prostate biopsy				
2003–2007	1.0			
2008–2013	0.71 (0.34–1.49)	0.364		
Number of biopsy cores taken				
≤ 12	1.0			
≥ 13	1.29 (0.60–2.76)	0.514		
Antibiotic prophylaxis				
Quinolone	1.0		1.0	
third-generation Cephalosporin	0.20 (0.10–0.42)	<0.001	0.21 (0.10–0.44)	<0.001

^a^Logistic regression analysis used to evaluate 5215 patients

Ten of the 15 patients with infectious complications who received prophylactic quinolone showed quinolone-resistant *Escherichia coli* in a blood culture, and three of this subgroup developed septic shock, that resulted in one death (Table 3). A lower rate of isolated bacteria, which was resistant to prophylactic antibiotics was observed in patients who were admitted after receiving prophylactic third-generation cephalosporin versus quinolone (3/13 [23.1 %] vs. 10/15 [66.7 %], *p* = 0.021). Interestingly, none of the patients who received prophylactic third-generation cephalosporin developed septic shock or died (Table 3). Eleven of 28 patients hospitalized due to high fever after TRUS-PBx showed no significant growth of bacteria in culture. However, the fever in these patients was controlled with conservative management including adequate administration of antibiotics.

**Table 3:** Clinical features of patients hospitalized due to infectious complications after prostate biopsy

Variable	Quinolone	Third generation cephalosporin
Number of patients (%)	15	13
Isolated bacteria (%)	73.3 (11/15)	46.2 (6/13)
*Escherichia coli*	10/11	1/6
*Enterococcus*	1/11	3/6
*Coagulase-negative staphylococcus*	0/11	1/6
*Klebsiella pneumoniae*	0/11	1/6
Septic shock (%)	20 (3/15)	0
Mortality (%)	6.7 (1/15)	0

## Discussion

Until innovative new technology can replace the TRUS-PBx in the future, this procedure will be performed several million times every year worldwide to diagnose prostate cancer. Several prevention strategies to reduce serious infectious complications after TRUS-PBx have been attempted to improve the safety of patients undergoing this procedure. Among the strategies, antibiotic prophylaxis can play an important key role in prevention of infection after TRUS-PBx. Current guidelines recommended the use of fluoroquinolones or cephalosporin (first generation, second generation, or third generation) before prostate biopsy [17]. Alternative antimicrobial regimens such as aminoglycosides plus metronidazole or clindamycin have been recommended [17]. In many studies, fluoroquinolones were used to examine empirical antimicrobial prophylaxis before prostate biopsy. In addition, fluoroquinolone-based antimicrobial prophylaxis is still used worldwide for standard antibiotic prophylaxis [18].

However, an increasing rate of infectious complications after TRUS-PBx due to the emergence of fluoroquinolone-resistant bacteria has recently been reported [12–14]. Adibi et al. showed that the addition of one dose of intramuscular gentamicin with ciprofloxacin reduced the rate of re-hospitalization due to infectious complications after prostate biopsy from 3.8 to 0.6 % [4]. Gianna et al. reported that a combination of orally administered quinolone and periprostatic injection of cephalosporin controls infection caused by fluoroquinolone-resistant *E.coli* [19]. The rates of rehospitalization after TRUS-PBx were 5.7 and 0 % in the conventional quinolone group and the combination group, respectively [19]. Bartus et al. reported that adding amikacin to fluoroquinolone-based antimicrobial prophylaxis confers a significant benefit in preventing infections after TRUS-guided biopsy [20]. In contrast, Hori et al. reported that ciprofloxacin provides superior prophylaxis compared to co-amoxiclav in men undergoing TRUS-PBx [21]. Although eight men (7.3 %) in the co-amoxiclav group developed sepsis, only two men in the ciprofloxacin group (1.7 %) developed sepsis after TRUS-PBx [21].

The prevalence of antimicrobial-resistant *E.coli* has changed in Korea, Lee et al. recently reported on data from the Korean antimicrobial resistance monitoring system between 2008 and 2009 that indicated *E.coli* isolates from cystitis patients had 75 % susceptibility to quinolone and 95 % susceptibility to third-generation cephalosporin [22]. Resistant *E.coli* rates also have also increased in other Asian countries [23]. Our study shows that use of third-generation cephalosporin before TRUS-PBx resulted in a significant decrease in infection-related hospitalization after TRUS-PBx. The pathogenesis of infectious complications after TRUS-PBx can be explained by the invasion of bacteria from the gastrointestinal tract, and cephalosporins are recommended as a prophylactic antibiotic in colorectal surgery [24]. The most appropriate regimen during TRUS-PBx is controversial. Our results showed that a regimen of prophylactic third-generation cephalosporin has a significant advantage in the context of preventing rehospitalization due to infectious complications after TRUS-PBx in an era of the emergence of quinolone-resistant microorganisms.

Interestingly, our data showed that severe symptoms on the IPSS questionnaire were significantly associated with hospitalization after TRUS-PBx. As lower urinary tract symptoms may partially indicate inflammation of the prostate and periprostatic tissue, TRUS-PBx could aggravate the inflammatory status of the prostate and result in a systemic inflammatory syndrome with high fever after prostate biopsy. Moreover, antibiotic-resistant bacteria increase following pre-administration of medication for prostatitis [12]. Mosharafa et al. reported that prior fluoroquinolone intake is a significant risk factor for acute prostatitis after TRUS-guided biopsy [14]. Additionally, the results of increased infection could be explained by bladder emptying which is known to be a very important factor in preventing urinary tract infection. High IPSS scores would be associated with inadequate bladder emptying, which could cause bacterial growth. In light of these findings, we consider the IPSS score in our current TRUS-PBx protocol, and recommend more stringent protocols for prophylaxis in men high IPSS scores.

Some controversy remains regarding the association between infection-related complications after prostate biopsy and procedure-related risk factors such as number of biopsy cores [6–8, 25]. Our study demonstrated that the number of biopsy cores was not associated with infection-related hospitalization after TRUS-PBx.

In the present study, we performed TRUS-PBx in an inpatient setting and we prescribed long-term use of antibiotics. However, the duration of administration of prophylactic antimicrobials remains controversial issues, even though a recent meta-analysis reported that long-term use of antibiotics (3 days) is not superior to short-course prophylactic (1 day) use in terms of symptomatic infections after prostate biopsy [18],. Furthermore, medical services depend not only on clinical guidelines but also patient preference or health insurance policies. Moreover, we thought that the reason for a low level of infectious hospitalization (0.54 %) may be due to our prophylactic management. Adibi et al. also demonstrated that a significant hospitalization for post-biopsy infectious complications from 3.8 % (11/290) to 0.6 % (2/310) after addition of gentamicin to quinolone [4].

The limitations of our study include its retrospective nature and the fact that the data were obtained from a single institution. Only 28 events of infection-related hospitalization after TRUS-PBx were observed in this cohort. This low number of events could compromise the accuracy of the model prediction. In addition, we could not assess specific information about the recent patient history of prior antibiotic use or urinary tract infection before TRUS-PBx. Also, because we studied data from a primarily inpatient only cohort, (only 5 % of patients underwent TRUS-PBx in the outpatient setting at our institute during the study period), our findings may not be applicable to patients in other setting undergoing TRUS-PBx. We focused on the effect of prophylactic antibiotics on infectious complications, thus we did not enroll out patients to avoid confounding. Furthermore, there may be an issue regarding the increasing risk of third generation cephalosporin resistance. Overall, we believe study so our findings warrant additional investigation using a randomized controlled trial.

## Conclusions

With the emergence of quinolone-resistant microorganisms, third-generation cephalosporin may effectively reduce the risk of infectious complications after TRUS-PBx. Severe lower urinary tract symptoms may also be an independent risk factor for infection-related hospitalization after TRUS-PBx. A large-scale, multicenter, prospective study is needed to fully evaluate the regimen of prophylactic antimicrobials in TRUS-PBx.

## Abbreviations

BMI: Body mass index
CI: Confidence interval
IPSS: International Prostate Symptom Score
OR: Odds ratios
PSA: Prostate specific antigen
TRUS-PBx: Transrectal ultrasound-guided prostate biopsy

## References

[1] Loeb S, Carter HB, Berndt SI, Ricker W, Schaeffer EM (2011). Complications after prostate biopsy: data from SEER-Medicare. J Urol.

[2] Loeb S, van den Heuvel S, Zhu X, Bangma CH, Schroder FH, Roobol MJ (2012). Infectious complications and hospital admissions after prostate biopsy in a European randomized trial. Eur Urol.

[3] Nam RK, Saskin R, Lee Y, Liu Y, Law C (2010). Increasing hospital admission rates for urological complications after transrectal ultrasound guided prostate biopsy. J Urol.

[4] Adibi M, Hornberger B, Bhat D, Raj G, Roehrborn CG, Lotan Y (2013). Reduction in hospital admission rates due to post-prostate biopsy infections after augmenting standard antibiotic prophylaxis. J Urol.

[5] Rosario, Lae JA, Metcalfe C, Donovan JL, Doble A (2012). Short term outcomes of prostate biopsy in men tested for cancer by prostate specific antigen: prospective evaluation within Protec T study. BMJ.

[6] Wagenlehner FM, van Oostrum E, Tenke P, Tandogdu Zm Cek M (2013). Infective complications after prostate biopsy: outcome of the Global Prevalence Study of Infections in Urology (GPIU) 2010 and 2011, a prospective multinational multicentre prostate biopsy study. Eur Urol.

[7] El-Hakim A, Moussa S (2010). CUA guidelines on prostate biopsy methodology. Can Urol Assoc J.

[8] Loeb S, Vellekoop A, Ahmed HU, Catto J, Emberton M, Nam R (2013). Systematic review of complications of prostate biopsy. Eur Urol.

[9] Wagenlehner FM, Pilatz A, Waliszewski P, Weidner W, Johansen TE (2014). Reducing infection rates after prostate biopsy. Nat Rev Urol.

[10] Heidenreich A, Bellmunt J, Bolla M, Joniau S, Manson M, Matveev V (2011). EAU guidelines on prostate cancer. Part 1: screening, diagnosis, and treatment of clinically localised disease. Eur Urol.

[11] Wolf JS, Bennett CJ, Dmochowski RR, Hollenbek BK, Pearle MS, Schaeffer AJ (2008). Best practice policy statement on urologic surgery antimicrobial prophylaxis. J Urol.

[12] Batura D, Rao GG, Nielsen PB (2010). Prevalence of antimicrobial resistance in intestinal flora of patients undergoing prostatic biopsy: implications for prophylaxis and treatment of infections after biopsy. BJU Int.

[13] Liss MA, Chang A, Santos R, Nakama-Peeples A, Peterson EM, Osann K (2011). Prevalence and significance of fluoroquinolone resistant Escherichia coli in patients undergoing transrectal ultrasound guided prostate needle biopsy. J Urol.

[14] Mosharafa AA, Torky MH, El Said WM, Mesherf A (2011). Rising incidence of acute prostatitis following prostate biopsy: fluoroquinolone resistance and exposure is a significant risk factor. Urology.

[15] Duplessis CA, Bavaro M, Simons MP, Marquet C, Santomauro M, Auqe B (2012). Rectal cultures before transrectal ultrasound-guided prostate biopsy reduce post-prostatic biopsy infection rates. Urology.

[16] Peabody JW, Lee SW, Bickel SR (1995). Health for all in the Republic of Korea: one country’s experience with impelmenting universal health care. Health Policy.

[17] Gonzalez CM, Averch T, Boyd LA, Clemens JQ, Dowling R, Goldman HB, et al. AUA/SUNA White Paper on the Incidence, Prevention and Treatment of Complications Related to Prostate Needle Biopsy. American Urological Association, 2012. https://www.auanet.org/common/pdf/practicesresources/quality/patient_safety/Prostate-Needle-Biopsy-White-Paper.pdf. Accessed 22 Aug 2016.

[18] Zani EL, Clark OA, Rodrigues Netto N (2011). Antibiotic prophylaxis for transrectal prostate biopsy. Cochrane Database Syst Rev.

[19] Pace G, Carmignani L, Marenghi C, Mombelli G, Bozzini G (2012). Cephalosporins periprostatic injection: are really effective on infections following prostate biopsy?. Int Urol Nephrol.

[20] Batura D, Rao GG, Bo Nielsen P, Charlett A (2011). Adding amikacin to fluoroquinolone-based antimicrobial prophylaxis reduces prostate biopsy infection rates. BJU Int.

[21] Hori S, Sengupta A, Joannides A, Baloqun-Ojuri B, Tilley R, McLoughlin J (2010). Changing antibiotic prophylaxis for transrectal ultrasound-guided prostate biopsies: are we putting our patients at risk?. BJU Int.

[22] Lee SJ, Lee DS, Choe HS, Shim BS, Kim CS, Kim ME (2011). Antimicrobial resistance in community-acquired urinary tract infections: results from the Korean Antimicrobial Resistance Monitoring System. J Infect Chemother.

[23] Sidjabat HE, Paterson DL (2015). Multidrug-resistant Escherichia coii in Asia: epidemiology and management. Expert Rev Anti Infect Ther.

[24] Bratzler DW, Houck PM (2005). Antimicrobial prophylaxis for surgery: an advisory statement from the National Surgical Infection Prevention Project. Am J Surg.

[25] Simsir A, Kismali E, Mammadov R, Gunaydin G, Cal C (2010). Is it possible to predict sepsis, the most serious complication in prostate biopsy?. Urol Int.

